# Миокиновый профиль у подростков с ожирением при аэробных физических нагрузках

**DOI:** 10.14341/probl13138

**Published:** 2022-07-24

**Authors:** Ю. В. Касьянова, О. В. Васюкова, П. Л. Окороков, З. Т. Зураева, О. Б. Безлепкина

**Affiliations:** Национальный медицинский исследовательский центр эндокринологии; Национальный медицинский исследовательский центр эндокринологии; Национальный медицинский исследовательский центр эндокринологии; Национальный медицинский исследовательский центр эндокринологии; Национальный медицинский исследовательский центр эндокринологии

**Keywords:** миокины, ожирение, дети, интерлейкин-6, миостатин, декорин, ирисин

## Abstract

**ОБОСНОВАНИЕ:**

ОБОСНОВАНИЕ. Миокины синтезируются миоцитами и высвобождаются в кровоток в ответ на сокращение мышечных волокон. Они оказывают положительное влияние на углеводный и липидный обмен, рост мышечной массы, остеогенез, повышают чувствительность тканей к инсулину, противодействуют воспалению жировой ткани. Изучение их секреции в ответ на физическую нагрузку (ФН) может помочь персонифицировать терапию ожирения.

**ЦЕЛЬ:**

ЦЕЛЬ. Изучить особенности секреции миокинов у детей с конституционально-экзогенным ожирением при физической нагрузке разной продолжительности и интенсивности и оценить их взаимосвязь с параметрами композиционного состава тела.

**МАТЕРИАЛЫ И МЕТОДЫ:**

МАТЕРИАЛЫ И МЕТОДЫ. В исследование включены 26 детей (10 мальчиков и 16 девочек) 15 [13; 16] лет, SDS ИМТ: +2,91 [2,24; 3,29], с половым развитием по Таннеру 4–5. Случайным распределением сформированы две группы по 13 человек. I группа выполняла ФН (ходьбу на беговой дорожке под контролем уровня частоты сердечных сокращений) разной продолжительности: 30 и 60 мин при одинаковой интенсивности (менее 3 метаболических эквивалентов (МЕТ)). II группа — ФН разной интенсивности: низкую — менее 3 МЕТ и умеренную — 3–6 МЕТ при одинаковой продолжительности 45 мин. Для определения уровня миокинов использованы коммерческие наборы для иммуноферментного анализа. Оценка композиционного состава тела проводилась методом биоимпедансного анализа (анализатор In Body 770, Южная Корея) утром, натощак. Статистическая обработка проводилась с применением STATISTICA v.12.0 (StatSoftInc., США). Результаты представлены в виде медианы (Ме) и квартилей (Q1; Q3), соответствующих 25 и 75 перцентилям. Критический уровень значимости (р) принимали равным <0,05.

**РЕЗУЛЬТАТЫ:**

РЕЗУЛЬТАТЫ. ФН умеренной интенсивности приводит к максимальному повышению уровня миокинов: интерлейкина-6 (ИЛ-6) на 215,7% и декорина на 34,3%, снижению уровня ирисина на 16,5%. Часовая тренировка низкой интенсивности приводит к умеренному повышению уровня ИЛ-6 на 80,5%, снижению уровня ирисина на 31,1%. Миостатин одинаково повышается как после 60-минутной ФН, так и после ФН умеренной интенсивности на 30,9 и 31,8% соответственно. Короткая низкоинтенсивная ФН (продолжительностью 30 мин) не сопровождается значимым повышением экспрессии миокинов. Отмечена взаимосвязь между количеством мышечной (r=0,65), тощей (r=0,62), безжировой массы (r=0,64) и уровнем декорина после ФН. Статистически значимой взаимосвязи между параметрами композиционного состава тела и уровнями ИЛ-6, миостатина, ирисина не выявлено. Гендерных различий как в базальной, так и в стимулированной секреции миокинов не выявлено.

**ЗАКЛЮЧЕНИЕ:**

ЗАКЛЮЧЕНИЕ. ФН умеренной интенсивности и 60-минутная ФН низкой интенсивности наиболее эффективны для детей с ожирением. 30-минутная ФН низкой интенсивности недостаточна для повышения секреции миокинов скелетной мускулатурой.

## Abstract (en)

**BACKGROUND:**

BACKGROUND: Myokines are synthesized by myocytes and released into the bloodstream in response to muscle fiber contraction. They have a positive effect on carbohydrate and lipid metabolism, muscle mass growth, osteogenesis, increase tissue sensitivity to insulin, counteract inflammation of adipose tissue. The study of their secretion in response to physical activity (FA) can help to personalize the therapy of obesity.

**AIM:**

AIM: to study the features of the secretion of myokines in children with constitutionally exogenous obesity during physical activity of different duration and intensity and to evaluate their relationship with the parameters of the body composition.

**MATERIALS AND METHODS:**

MATERIALS AND METHODS: 26 children (10 boys and 16 girls) were included in the study 15 [13; 16] years old, SDS BMI: +2.91 [2.24; 3.29], with sexual development according to Tanner 4–5. Two groups of 13 people were formed by random distribution. Group I performed FA (walking on a treadmill under the control of heart rate) of different duration: 30 and 60 minutes at the same intensity (less than 3 metabolic equivalents (MET)). Group II — FA of different intensity: low — less than 3 METH and moderate — 3–6 METH with the same duration of 45 minutes. Commercial kits for enzyme immunoassay were used to determine the level of myokines. The assessment of the compositional composition of the body was carried out by bioimpedance analysis (analyzer In Body 770, South Korea) in the morning, on an empty stomach. Statistical processing was carried out using STATISTICA v.12.0 (StatSoftInc., USA). The results are presented in the form of median (Me) and quartiles (Q1; Q3) corresponding to 25 and 75 percentiles. The critical significance level (p) was assumed to be <0.05.

**RESULTS:**

RESULTS: moderate intensity FA leads to a maximum increase in the level of myokines: interleukin-6 (IL-6) by 215.7% and decorin by 34.3%, a decrease in the level of irisin by 16.5%. An hour-long low-intensity workout leads to a moderate increase in the level of IL-6 by 80.5%, to a decrease in the level of irisin by 31.1%. Myostatin increases equally both after 60-minute FA and after moderate intensity FA by 30.9% and 31.8%, respectively. Short low-intensity FA (lasting 30 minutes) it is not accompanied by a significant increase in the expression of myokines. The relationship between the amount of muscle (r=0.65), lean (r=0.62), fat-free mass (r=0.64) and the level of decorin after FA was noted. There was no statistically significant relationship between the parameters of the body composition and the levels of IL-6, myostatin, and irisin. There were no gender differences in both basal and stimulated myokine secretion.

**CONCLUSION:**

CONCLUSION: Moderate intensity FA and low intensity 60-minute FA are most effective for obese children. A 30-minute low-intensity FN is insufficient to increase the secretion of myokines by skeletal muscles.

## ОБОСНОВАНИЕ

Исследования последних лет показали, что сокращающиеся скелетные мышцы могут секретировать и высвобождать в системный кровоток молекулы, названные миокинами. К ним относятся интерлейкин-6 (ИЛ-6), ирисин, миостатин, декорин, интерлейкин-15 (ИЛ-15), нейротрофический фактор головного мозга (BDNF), b-аминоизомасляная кислота, фактор роста фибробластов-21 (FGF-21) и другие. Помимо мышечной ткани, часть из них может синтезироваться другими органами и тканями, например, FGF-21 — печенью; ирисин, ИЛ-15, миостатин — жировой тканью; в связи с чем они классифицируются как гепатокины и адипомиокины. Однако их общей особенностью является повышение секреции в ответ на физические упражнения.

Под влиянием физической нагрузки происходят сложные биохимические реакции в мышечных волокнах на клеточном и молекулярном уровне (изменяется активность ферментов, способность к окислению различных энергетических субстратов), а также изменения физиологических показателей организма (увеличение сердечного выброса и легочной вентиляции). Метаболические сдвиги в мышечной ткани при активной физической деятельности связаны с повышенным усвоением глюкозы и жирных кислот — главных источников энергии. Ввиду ограниченного содержания в мышечной ткани, их основными поставщиками становятся печень, в которой активируется распад гликогена, и жировая ткань, в которой повышается синтез жирных кислот. Реализация данных физиологических эффектов при физической нагрузке (ФН) связана с повышением чувствительности к инсулину в мышечных клетках и снижением в гепатоцитах и адипоцитах. Ключевым звеном, ответственным за развитие противоположных метаболических сдвигов, является ИЛ-6.

ИЛ-6 — один из первых цитокинов, классифицированных как миокин [1]. Его основная биологическая роль во время ФН заключается в обеспечении сокращающихся мышц энергетическим субстратом, главным образом глюкозой. Он стимулирует повышение гликогенолиза в печени, увеличивает экспрессию транспортера глюкозы 4 типа (GLUD-4) и поглощение глюкозы в миоцитах, повышает их чувствительность к инсулину. Вместе с тем в адипоцитах ИЛ-6 стимулирует липолиз и окисление свободных жирных кислот, которые также используются миоцитами как источник энергии. Помимо этого, он тормозит инсулинопосредованное поглощение глюкозы адипоцитами и гликогенез в печени.

Ирисин — адипомиокин, секретирующийся клетками как в мышечной, так и в жировой ткани. Ирисин положительно влияет на углеводный и липидный обмен, повышая толерантность к глюкозе и снижая инсулинорезистентность, стимулируя липолиз иингибируя липогенез [2]. Помимо этого, оказывает противовоспалительное действие в адипоцитах, макрофагах, гепатоцитах, способствует повышению минеральной плотности костной ткани [3].

Миостатин относится к семейству трансформирующего фактора роста β, является мощным ингибитором гипертрофии мышечной ткани, участвует в дифференцировке и пролиферации миосателлитов, синтезе белка, процессе адаптации мышц к физическим нагрузкам [4]. Помимо этого, он способствует резорбции костной ткани, адипогенезу, формированию инсулинорезистентности. Декорин является его прямым антагонистом, способствуя гипертрофии скелетных мышц путем связывания миостатина и регулируя рост мышц при физической нагрузке.

Не вызывает сомнений, что физические упражнения оказывают благоприятное влияние на улучшение метаболических нарушений и являются неотъемлемой частью стратегии в борьбе с ожирением, в профилактике хронических неинфекционных заболеваний. Изучение миокинов расширяет понимание того, как мышцы взаимодействуют с другими органами и тканями, такими как жировая и костная ткань, печень, поджелудочная железа, головной мозг, а величина высвобождения миокинов в кровь в ответ на мышечные сокращения может помочь объяснить преимущества ФН для организма. Однако влияние интенсивности и продолжительности нагрузки, а также вида физических упражнений на изменение секреции миокинов остается малоизученным. Также неизвестны особенности влияния композиционного состава тела на экспрессию миокинов при ожирении в условиях малоподвижного образа жизни. И наконец, исследования миокинов у детей с ожирением единичны ипротиворечивы.

## ЦЕЛЬ ИССЛЕДОВАНИЯ

Изучить особенности секреции миокинов у детей с конституционально-экзогенным ожирением при физической нагрузке разной продолжительности и интенсивности, оценить их взаимосвязь с параметрами композиционного состава тела.

## МАТЕРИАЛЫ И МЕТОДЫ

## Дизайн исследования

Проведено обсервационное одноцентровое одномоментное выборочное контролируемое исследование.

## Критерии соответствия

В исследование включены 26 пациентов (10 мальчиков и 16 девочек) от 12 до 17,9 года с конституционально-экзогенным ожирением, SDS ИМТ≥+2,0 (SDS — от англ. standart deviation score — коэффициент стандартного отклонения; ИМТ — индекс массы тела), с пубертатным половым развитием (стадия 4–5 по Таннеру).

Критериями исключения из исследования были пациенты, получавшие медикаментозную терапию ожирения на момент обследования, с другими формами ожирения (гипоталамическое, синдромальное и др.) и тяжелыми сопутствующими заболеваниями (ортопедическая патология, заболевание легочной системы, некомпенсированная артериальная гипертензия, психические расстройства).

## Место и время проведения исследования

Место проведения. Исследование проводилось на базе ФГБУ «НМИЦ эндокринологии» Минздрава РФ в детском отделении тиреоидологии, репродуктивного и соматического развития.

Время исследования. Включение пациентов в исследование проведено за 2021 г. Образцы сывороток исследовались одномоментно после сбора всего биологического материала.

## Описание медицинского вмешательства

Антропометрические измерения включали: измерение роста, веса, расчет ИМТ. ИМТ оценивался по нормативам для конкретного возраста и пола и представлен в виде числа стандартных отклонений от среднего (SDS). Оценка полового развития проводилась по классификации Таннера. Всем пациентам выполнена оценка композиционного состава тела. Исследование уровней миокинов в сыворотке крови проводилось утром натощак (в покое) и после физической нагрузки.

## Исходы исследования

Основной исход исследования: определены сывороточные уровни миокинов — миостатина, декорина, ирисина, ИЛ-6 в покое и после физической нагрузки разной продолжительности и интенсивности.

Дополнительные исходы исследования: определены и изучены особенности композиционного состава тела — процентное содержание жировой ткани, тощей массы, скелетно-мышечной ткани.

## Анализ в подгруппах

Случайным распределением сформированы две группы. Пациенты 1-й группы (13 человек) выполняли ФН (ходьбу на беговой дорожке под контролем уровня частоты сердечных сокращений) разной продолжительности: 30 и 60 мин при одинаковой интенсивности (менее 3 метаболических эквивалентов (МЕТ)). У пациентов 2-й группы (13 человек) ФН имела разную интенсивность: низкую — менее 3 МЕТ и умеренную — 3–6 МЕТ при одинаковой продолжительности в течение 45 мин.

## Методы регистрации исходов

Значение SDS ИМТ≥2,0 считали критерием ожирения (согласно рекомендациям ВОЗ и национальным рекомендациям).

Лабораторные исследования проводились в клинико-диагностической лаборатории ФГБУ «НМИЦ эндокринологии» Минздрава России. Концентрация миокинов в сыворотке крови определялась наборами для иммуноферментного анализа: ирисин — Biovendor, ИЛ-6 –Bender Medsystems, декорин — Aviscera Bioscience, миостатин — ELISA kit Immundiagnostik AG. Степень повышения миокинов представлена в виде доли в процентах от исходных значений (полученных в покое), вычислена по формуле:

% = (B-A)/A×100 (A = исходное значение, B = конечное значение).

Оценка композиционного состава тела проводилась методом биоимпедансного анализа (анализатор In Body 770, Южная Корея) утром, натощак. Определялось содержание жировой массы (%), безжировой и тощей массы (кг), мышечной массы (кг).

Нагрузочную калориметрию проводили с помощью метаболографа Quark RMR (Италия), подключенного к велоэргометру (Lode, Германия). Исследование проводилось спустя 2 ч после еды. Использовался стандартизированный протокол ФН спостоянным ступенчатым повышением мощности под контролем частоты сердечных сокращений с помощью нагрудного пульсометра Polar (Финляндия). Расчет максимального уровня потребления кислорода (VO2max) не проводился.

Согласно рекомендациям Всемирной организации здоровья (ВОЗ), для выражения степени интенсивности физической активности использовался метаболический эквивалент (МЕТ). МЕТ — это отношение уровня метаболизма человека во время физической активности к уровню его метаболизма в состоянии покоя. Один МЕТ — это количество энергии, затрачиваемое человеком в состоянии покоя и эквивалентное сжиганию 1 ккал/кг/ч или потребляемое организмом количество кислорода в состоянии покоя, равное 3,5 мл О2 на 1 кг массы тела в минуту. По сравнению с состоянием покоя человек при умеренной физической активности сжигает в 3–6 раз больше калорий (3–6 МЕТ), а при высокой — более чем в 6 раз (>6 МЕТ).

## Этическая экспертиза

Протокол исследования одобрен 12.02.2020 локальным Этическим комитетом при ФГБУ «Национальный медицинский исследовательский центр эндокринологии» Минздрава России (выписка из протокола №2 от 12.02.2020 г.). Родители всех включенных пациентов подписали добровольное информированное согласие на участие в исследовании.

## Статистический анализ

Статистическая обработка данных проводилась с использованием Excel 2016 (Microsoft, USA), программы Statistica (версии 12.0, StatSoft Inc., США). Результаты представлены в виде медианы (Ме) и квартилей (Q1; Q3), соответствующих 25 и 75 перцентилям. Для оценки изменений уровня миокинов после ФН использовался ­критерий Уилкоксона. Для оценки достоверности различий между изучаемыми подгруппами использовались критерий Манна–Уитни и дисперсионный анализ Краскела–Уоллеса. Корреляционный анализ проводился с использованием критерия Спирмена. Критический уровень значимости различий принимали ≤0,05.

## РЕЗУЛЬТАТЫ

## Клинико-антропометрическая характеристика обследованных пациентов

Обследовано 26 подростков, 10 мальчиков (38%) и 16 девочек (62%), 15 [ 13; 16] лет, с половым развитием по Таннеру 4–5.

Конституционально-экзогенное ожирение I степени было у 8 пациентов (31%), 7 из которых девочки, II степени — у 6 (23 %) и III степени — у 12 (46 %), в равных количествах у обоих полов. Таким образом, в исследуемой группе преобладали подростки с выраженным ожирением, SDS ИМТ: +2,91 [ 2,24; 3,29].

По данным биоимпедансометрии у всех обследованных пациентов содержание жировой ткани в организме составило 40 [ 36,5; 44,0]% (36,35 [ 30,7;42,6] кг, избыток жировой ткани — 14,2 [ 8,9;21,2] кг, масса скелетной мускулатуры, тощая и безжировая масса — 30,55 [ 26,9; 35,7] кг, 49,65 [ 45,1; 58,6] кг и 53,0 [ 48,0; 62,2] кг соответственно.

Анализируя гендерные различия, у мальчиков по сравнению с девочками показатели роста и веса были статистически значимо выше 177,2 [ 168,4; 185] см vs 167,5 [ 162,5; 170,3] см (р=0,019) и 99,25 [ 93,0; 106,0] кг vs 88,3 [ 81,55; 96,0] кг (р=0,027) соответственно, при сопоставимых показателях SDS ИМТ. Также у мальчиков отмечалось статистически значимо большее содержание в организме тощей массы — 59,1 [ 49,4; 62,3] vs 46,3 [ 43,65; 50,3] кг (р=0,0066) и безжировой массы 62,75 [ 52,5; 52,5] vs 51,1 [ 47,3; 54,85] кг (р=0,013) (табл. 1).

**Table:** Таблица 1. Гендерные различия исследуемых показателейTable 1. Gender differences in the studied indicators Данные представлены в виде медианы с интерквартильным интервалом: Ме [ Q1; Q3].SDS — стандартное отклонение от среднего; СММ, кг — скелетная мышечная масса; ОО, ккал/сут — основной обмен; ИЛ-6 — интерлейкин -6.

	Мальчики	Девочки	Р
Возраст, лет	13,5 [ 13,0; 15]	15[ 14,0; 16]	0,225
Рост, см	177,2 [ 168,4; 185]	167,5 [ 162,5; 170,3]	0,19
Вес, кг	99,25 [ 93,0; 106,0]	88,3 [ 81,55; 96,0]	0,027
SDS ИМТ	3,1 [ 2,78; 3,45]	2,87 [ 2,22; 3,18]	0,16
% жировой ткани	38, 6 [ 34,9; 42,7]	40,75 [ 38,2; 45,1]	0,28
Жировая ткань, кг	38,2 [ 33,2; 43,1]	34,7 [ 29,65; 41,5]	0,34
Тощая масса, кг	59,1 [ 49,4; 62,3]	46,3 [ 43,65; 50,3]	0,0066
Безжировая масса, кг	62,75 [ 52,5;52,5]	51,1 [ 47,3; 54,85]	0,013
СММ, кг	35,3 [ 29,1; 37,3]	29,3 [ 26,7; 3,85]	0,092
ИЛ-6	0,215 [ 0,162; 0,391]	0,204 [ 0,102; 0,305]	0,3
Миостатин	22,02 [ 20,1; 24,36]	22,12 [ 19,98; 25,45]	0,81
Декорин	4043,0 [ 3441,5; 4499,2]	4103,7 [ 3804,5; 4476,4]	1,0
Ирисин	25,28 [ 19,97; 34,8]	34,06 [ 25,22; 36,15]	0,27

## Уровни миокинов в сыворотке крови

В покое у всех обследованных пациентов концентрация ИЛ-6 составила 0,215 [ 0,16; 0,33] пг/мл, миостатина 22,12 [ 20,08; 25,04] нг/мл, декорина 4066 [ 3751; 4484] нг/мл, ирисина 32,78 [ 22,27; 35,95] мкг/мл. При этом, несмотря на большее содержание тощей массы у мальчиков, гендерных различий в уровнях миокинов в покое выявлено не было (см. табл. 1).

## Динамика уровня миокинов в исследуемых группах

Перед проведением ФН пациенты были разделены на две группы. В каждую были включены по 13 пациентов (5 мальчиков, 8 девочек) в возрасте 14,6 [ 14; 15] и 14,5 [ 13; 16] года, с SDS ИМТ 2,8 [ 2,2; 3,2] и 2,9 [ 2,7; 3,3] соответственно. Группы были сопоставимы по возрасту, полу, стадии полового развития, антропометрическим параметрам, степени ожирения, выраженной в SDS ИМТ, уровню миокинов. Клинические характеристики представлены в таблице 2.

**Table:** Таблица 2. Клиническая характеристика исследуемых группTable 2. Clinical characteristics of the study groups Данные представлены в виде медианы с интерквартильным интервалом: Ме [ Q1;Q3].SDS — стандартное отклонение от среднего.

	1-я группа	2-я группа	Р
Количество пациентов, n	13	13	>0.05
Возраст, лет	14,6 [ 14; 15]	14,5 [ 13; 16]	>0,05
Пол м/ж	5/8	5/8	>0,05
Рост, см	168,6 [ 164; 176,2]	169 [ 164,1; 174,7]	>0,05
Вес, кг	90 [ 84,8; 100,7]	93 [ 88; 99]	>0,05
SDS ИМТ	2,8 [ 2,2; 3,2]	2,9 [ 2,7; 3,3]	>0,05
Стадия полового развития, Таннер	4–5	4–5	>0,05
% жировой ткани	39,2 [ 34,9; 44,0]	41,6 [ 38,4; 43,2]	>0,05
Жировая ткань, кг	36,2 [ 29,4; 41,8]	38,5 [ 32,6; 42,6]	>0,05
Тощая масса, кг	46,7 [ 43,5; 58,7]	50,3 [ 45,9; 53,3]	>0,05
Безжировая масса, кг	52,6 [ 48,0; 62,4]	52,3 [ 48,6; 56,9]	>0,05
СММ, кг	31,6 [ 26,5; 35,7]	30,1 [ 29,1; 34,9]	>0,05
Миостатин, нг/мл	20,19 [ 18,65; 23,06]	23,94 [ 21,53; 25,85]	>0,05
Декорин, пг/мл	3891 [ 3036; 4469]	4332 [ 4043; 4743]	>0,05
ИЛ-6, пг/мл	0,226 [ 0,162; 0,246]	0,204 [ 0,12; 0,356]	>0,05
Ирисин, мкг/мл	33,32 [ 22,516; 36,348]	31,896 [ 22,27; 34,8]	>0,05

Первая группа выполняла ФН легкой интенсивности (ЛИФН) при разной продолжительности, 30 и 60 мин.

30-минутная ФН значимо не влияла на изменение концентрации миокинов.

После 60 мин ФН уровень ИЛ-6 статистически значимо увеличился с 0,226 [ 0,162; 0,246] пг/мл до 0,408 [ 0,279; 0,447] пг/мл (р=0,005) — на 80,5% по сравнению с базальным.

Было отмечено статически значимое повышение уровня миостатина после 60 мин ФН с 20,19 [ 18,65; 23,06] нг/мл до 26,62 [ 20,71; 28,48] нг/мл (р=0,0046), на 31,8%.

При этом значимых изменений уровня декорина после обеих видов нагрузок выявлено не было, но была отмечена тенденция к повышению его уровня.

Ирисин после 60 мин ФН статистически значимо снизился с 33,32 [ 22,52; 36,35] мкг/мл до 23,0 [ 18,52; 28,61] мкг/мл (р=0,003), на 31,1%.

Таким образом, 60-минутная продолжительность ФН сопровождалась более выраженным изменением уровня миокинов по сравнению с 30-минутной (табл. 3).

**Table:** Таблица 3. Содержание миокинов сыворотки крови после физических нагрузок разной продолжительностиTable 3. The content of myokines in blood serum after exercise of various durations

	Базально (1)	30 мин ФН (2)	60 мин ФН (3)	Р≤1, 2	Р≤1, 3
ИЛ-6, пг/мл	0,226 [ 0,162; 0,246]	0,327 [ 0,226; 0,403]	0,408 [ 0,279; 0,447]	0,06	0,005
Миостатин, нг/мл	20,19 [ 18,65; 23,06]	23,59 [ 22,31; 26,23]	26,62 [ 20,71; 28,48]	0,116	0,005
Декорин, пг/мл	3892 [ 3036; 4469]	4179 [ 3859; 4713]	4088 [ 3381; 4882]	0,075	0,096
Ирисин, мкг/мл	33,32 [ 22,52; 36,35]	25,1 [ 23,78; 32,24]	23,0 [ 18,52; 28,61]	0,116	0,003

Вторая группа выполняла ЛИФН (менее 3 МЕТ) и умеренной (3–6 МЕТ) интенсивности (УИФН) при одинаковой продолжительности 45 мин.

Уровень ИЛ-6 статистически значимо увеличился как после ЛИФН, так и после УИФН при равной продолжительности 45 мин, с 0,204 [ 0,12; 0,356] пг/мл до 0,485 [ 0,268; 0,748] пг/мл (р=0,0077) и 0,644 [ 0,282; 0,736] пг/мл (р=0,0044), на 137,8 и 215,7% соответственно.

Концентрация миостатина статистически значимо возросла после обеих нагрузок с 23,94 [ 21,53; 25,85] нг/мл до 27,94 [ 24,85; 28,56] нг/мл (р=0,0088) и 31,33 [ 24,67; 32,31] нг/мл (р=0,0028), на 16,7 и 30,9% соответственно.

Также отмечено статистически значимое повышение уровня декорина с 4332 [ 4043; 4743] пг/мл до 5075 [ 4058; 5576] пг/мл (р=0,013) и 5819 [ 4533; 5866] пг/мл (р=0,0088), на 17,2 и 34,3% соответственно.

Была отмечена тенденция к снижению уровня ирисина после УИФН, статистически значимое снижение выявлено после ЛИФН с 32,1 [ 22,27; 34,8] мкг/мл до 26,8 [ 22,76; 28,3] мкг/мл (р=0,046), на 16,5%.

Таким образом, увеличение интенсивности ФН приводило к более значимому увеличению концентрации ИЛ-6, миостатина и декорина (табл. 4).

**Table:** Таблица 4. Содержание миокинов сыворотки крови после физических нагрузок разной интенсивностиTable 4. Content of blood serum myokines after physical activity of different intensity

	Базально (1)	45 мин ЛИФН (2)	45 мин УИФН (3)	Р≤1, 2	Р≤1, 3
ИЛ-6, пг/мл	0.204 [ 0,12; 0,356]	0,485 [ 0,268; 0,748]	0,644 [ 0,282; 0,736]	0,0077	0,0044
Миостатин, нг/мл	23.94 [ 21,53; 25,85]	27,94 [ 24,85; 28,56]	31,33 [ 24,67; 32,31]	0,0088	0,0028
Декорин, пг/мл	4332 [ 4043; 4743]	5075 [ 4058; 5576]	5819 [ 4533; 5866]	0,013	0,0088
Ирисин, мкг/мл	32,1 [ 22,27; 34,8]	26,8 [ 22,76; 28,3]	25,1 [ 18,12; 31,55]	0,046	0,0546

Сравнение исследуемых групп

При сравнении изменений уровней миокинов между группами разной продолжительности и интенсивности ФН концентрация миостатина была статистически значимо выше во 2-й группе при УИФН, чем в 1-й при ФН длительностью 30 мин (р=0,0355), что также отмечено в отношении ИЛ-6 (р=0,068965). Наиболее высокий уровень декорина выявлен у подростков 2-й группы, выполнявших УИФН по сравнению с детьми 1-й группы при ФН продолжительностью 30 мин (р=0,0096) и 60 мин (р=0,011978).

Таким образом, умеренные нагрузки способствуют более высокой концентрации миокинов в сыворотке крови (табл. 5).

**Table:** Таблица 5. Сравнение физических нагрузок разной продолжительности и интенсивностиTable 5. Comparison of physical activity of different duration and intensity Данные представлены в виде р-value.

	р-value
ИЛ-6	миостатин	декорин	ирисин
30 мин — ЛИФН	0,248536	0,111894	0,123937	0,608077
30 мин — УИФН	0,068965	0,035505	0,009605	0,959101
60 мин — ЛИФН	0,355939	0,383321	0,081232	0,719615
60 мин — УИФН	0,165858	0,072675	0,011978	0,521506

Корреляционный анализ

При проведении корреляционного анализа между концентрацией миокинов в сыворотке крови в покое, антропометрическими показателями и параметрами композиционного состава тела выявлена положительная взаимосвязь уровня декорина с количеством мышечной (r=0,57), тощей (r=0,59) и безжировой массы (r=0,75).

Значимого влияния исследуемых показателей на уровень остальных миокинов в покое выявлено не было.

Положительная корреляционная связь между концентрацией декорина с количеством мышечной (r=0,65), тощей (r=0,62) и безжировой массы (r=0,64) также сохранялась при умеренной интенсивной ФН.

Можно предположить, что это связано с большей мышечной работой, совершаемой при более интенсивной ФН. Также нельзя исключить вклад повседневной физической активности (наличие регулярных ФН) и выносливости каждого пациента, что может оказывать большее влияние на стимулированную секрецию миокинов и функциональную активность мышечной ткани в целом, а не определяться просто количеством мышечной массы. Помимо этого, стоит помнить, что миокины экспрессируются также жировой тканью, а выраженный ее избыток при ожирении может модулировать секрецию миокинов.

## ОБСУЖДЕНИЕ

В нашем исследовании мы получили статистически значимое повышение уровня ИЛ-6 при продолжительной (60-минутной) физической нагрузке — на 80,5%, а также ФН разной интенсивности: легкой на 137,8% и умеренной на 215,7% (при одинаковой длительности нагрузки 45 мин). Таким образом, максимальные значения ИЛ-6 отмечались при повышении интенсивности тренировки, а ФН продолжительностью 30 мин была недостаточной для значимого увеличения уровня ИЛ-6.

Повышение уровня ИЛ-6 в ответ на разные виды ФН как у лиц с нормальной массой тела, так и с ожирением показано в многочисленных работах [5–6][8], что согласуется с результатами, полученными в нашем исследовании. Важно отметить, что степень повышения ИЛ-6 зависит от продолжительности упражнений, а также количества мышечной массы, участвующей в работе [9]. Кроме этого, важным фактором, определяющим степень повышения ИЛ-6 в сыворотке крови, является интенсивность тренировок, так как снижение запасов мышечного гликогена и накопление лактата стимулирует его высвобождение из сокращающихся мышечных волокон [10]. Отсутствие в нашем исследовании корреляционных связей между повышением уровня ИЛ-6 и количеством мышечной массы может объясняться как небольшой выборкой пациентов, так и свидетельствовать о нарушении функциональной активности скелетной мускулатуры у детей с ожирением.

В нашей работе установлено статистически значимое повышение уровня миостатина — как при увеличении продолжительности ФН (с 30 до 60 мин) на 31,8%, так и при увеличении интенсивности ФН (с легкой до умеренной) на 30,9% по сравнению с базальной концентрацией. Таким образом, оба вида ФН привели к равному повышению уровня миостатина. Стоит отметить, что работ о влиянии ФН на уровень миостатина у детей как в отечественной, так и в зарубежной литературе до настоящего времени не опубликовано.

В ряде исследований у взрослых пациентов также отмечено повышение уровня миостатина в сыворотке крови сразу после ФН. B. Kabak и соавт. (2018 г.) изучали влияние высокоинтенсивных интервальных тренировок на уровень миостатина в плазме крови у кикбоксеров и мужчин, ведущих малоподвижный образ жизни, с нормальной массой тела, возрастом от 18 до 24 лет [11]. Концентрации миостатина в покое были сопоставимы в обеих группах, значимо увеличились сразу после тренировки (р<0,05), а через 3 ч снизились до исходного уровня. Подобный результат был продемонстрирован K. Kerschan-Schindl и соавт. (2015 г.) после ультрамарафона (246 км), где уровень миостатина был значимо выше после бега [12]. Авторами сделан вывод, что повышение миостатина в сыворотке крови, по-видимому, отражает катаболические процессы в мышцах, вызванные чрезмерной ФН и их повреждением. Одной из гипотез данного состояния является взаимосвязь с развитием воспаления, которое сопровождается высвобождением других цитокинов, ответственных за синтез миостатина. В работе Z. He и соавт. также отмечено повышение уровня миостатина сразу после ФН (в течение первых 3 ч), выполняемой на уровне аэробного порога, и последующее его снижение с достижением исходных значений через 72 ч после тренировки [13]. Авторы предполагают, что увеличение миостатина не обязательно должно приводить к снижению гипертрофии скелетных мышц и анаболической реакции на физические упражнения. Ввиду того, что основной функцией миостатина считается подавление роста и дифференцировки мышечной ткани, ожидалось снижение его концентрации. Однако на данный момент однозначного объяснения его транзиторного повышения в ответ на ФН не получено.

В нашей работе мы выявили тенденцию к снижению концентрации ирисина как после ФН разной продолжительности, так и интенсивности, но наиболее выраженное снижение отмечено через 60 мин ФН — на 23,3% по сравнению с базальным значением (р=0,003).

По данным других работ отмечались противоречивые результаты, наблюдалось как снижение [14], так и повышение [15], а в ряде исследований отсутствовали статистически значимые изменения.

В исследовании R. Lagzdina и соавт., включившем 84 здоровых взрослых 20–50 лет (40 мужчин, 44 женщины), после ФН у 58% участников не было выявлено статистически значимого изменения концентрации ирисина после однократной аэробной ФН,в то время как у 23% его уровень уменьшился, а у 19% увеличился [7].

Интересный дизайн представлен в работе D. Löffler и соавт. [15]. Исследователи проанализировали гендерные различия у детей и взрослых, а также сравнили динамику уровня ирисина у лиц с нормальной массой тела и с ожирением. Согласно результатам, у всех детей после 15-минутного теста с нагрузкой максимальной интенсивности уровень ирисина сыворотки повышался в среднем на 123%, при этом гендерных различий выявлено не было. У 70% взрослых, включивших мужчин без избытка массы тела и женщин с ожирением, статистически значимое повышение уровня ирисина отмечалось после 30 мин высокоинтенсивной ФН, что не было выявлено у худых женщин и мужчин с ожирением. При этом повышенный уровень ирисина отмечается непосредственно сразу после тренировки и снижался через 30 мин после. В исследовании отмечено, что концентрация ирисина в сыворотке положительно коррелировала с количеством безжировой массы (r=0,60, р=0,002) у взрослых, тогда как у детей подобной взаимосвязи выявлено не было. Также были выявлены гендерные различия: более высокий уровень ирисина в покое у мужчин по сравнению с женщинами, что не было отмечено у детей, хотя у девочек без избытка веса его уровень был несколько выше.

В нашей работе также не выявлено гендерных различий по уровню ирисина, что согласуется с данными S. Blüher и соавт. [16], тогда как в работе N.M. Al-Daghri и соавт. у девочек уровень ирисина был статистически значимо выше (р=0,04) [17].

Следует учитывать, что состав тела у детей отличается от взрослых. Кроме того, ирисин секретируется как миоцитами, так и адипоцитами, а выраженный избыток жировой ткани при ожирении, вероятно, может негативно влиять на секрецию данного миокина в ответ на ФН. Таким образом, вопрос о том, связан ли уровень ирисина с жировой и/или мышечной массой, в настоящее время остается предметом дискуссий.

Резюмируя вышеизложенное, можно сказать, что уровень ирисина после однократной ФН может быть различным как у детей, так и взрослых при разном ИМТ.

Таким образом, необходимы дальнейшие исследования, чтобы подтвердить влияние физических упражнений на концентрацию ирисина.

В нашем исследовании после УИФН отмечалось статистически значимое повышение уровня декорина на 34,3% (р=0,0088).

Среди немногочисленных исследований по изучению декорина у взрослых также выявлено повышение его концентрации сразу после ФН с последующим постепенным снижением. Так, в работе T. Kanzleiter и соавт., включившей 8 здоровых спортсменов, после силовой тренировки уровень декорина статически значимо повысился на 30% исходного, при этом у лиц с более высокой силовой выносливостью отмечался более выраженный подъем концентрации после ФН [18]. В исследовании P. Knuiman и соавт., включившем 13 молодых мужчин, которым сначала проводили тренировку на выносливость, а через 4 ч отдыха — силовую тренировку, статистически значимого повышения уровня декорина не зафиксировано, но отмечена тенденция к его повышению [19]. Однако в данной работе забор крови непосредственно после тренировки, когда отмечается максимальный уровень миокина, не проводился, что может объяснять полученный результат. Наконец, в исследовании E.M. Bugera и соавт. уровень декорина, определенный сразу после силовой ФН низкой и высокой интенсивности, был на 11,91% выше по сравнению с его концентрацией в покое, чем через 1 и 24 ч после тренировки, при этом различий между видами нагрузок выявлено не было [20].

Таким образом, несмотря на неоднозначные результаты различных исследований, изучение секреции миокинов у детей с конституционально-экзогенным ожирением является важным для понимания взаимодействия мышечной и жировой ткани. Требуется дальнейшее исследование миокинов на большей выборке пациентов с целью подтверждения описанных нами результатов, а также проведение оценки влияния уровня повседневной двигательной активности и имеющихся метаболических осложнений на секрецию миокинов.

## Ограничения исследования

Коллектив авторов допускает, что небольшой объем произведенной выборки с преобладающим количеством девочек в исследовании оказал определенное влияние на полученные результаты.

## ЗАКЛЮЧЕНИЕ

ФН у детей с конституционально-экзогенным ожирением сопровождается разнонаправленным изменением экспрессии миокинового профиля.

Увеличение интенсивности ФН сопровождается максимальным повышением уровня ИЛ-6, миостатина, декорина и уменьшением уровня ирисина.

Короткие низкоинтенсивные упражнения (до 30 мин) недостаточны для секреции сокращающимися скелетными мышцами значимого количества миокинов. Удлинение тренировки низкой интенсивности приводит к умеренному повышению уровня ИЛ-6 и миостатина, снижению уровня ирисина.

Таким образом, УИФН (3–6 МЕТ) в течение 45 минут и 60 минутные низкоинтенсивные физические упражнения наиболее предпочтительны для детей с ожирением.

Несмотря на гендерные различия композиционного состава тела, как базальная, так и стимулированная секреция миокинов у подростков разного пола статистически значимо не отличалась. Можно предположить, что особенности композиционного состава тела не позволяют спрогнозировать динамику уровня миокинов у детей с ожирением.

Поскольку основным источником секреции декорина является мышечная ткань, а при интенсивных ФН мышцы совершают большую работу, было выявлено влияние количества мышечной, тощей и безжировой массы на подъем уровня декорина после ФН.

Таким образом, изучение роли миокинов, особенностей их секреции является перспективным направлением для разработки персонифицированных рекомендаций по физической активности для детей с ожирением.

## ДОПОЛНИТЕЛЬНАЯ ИНФОРМАЦИЯ

Источник финансирования Публикация подготовлена в рамках госзадания «Новые подходы к персонифицированному лечению ожирения у детей на основе исследований энергетического обмена, функционального резерва бета-клеток, секреции адипокинов, миокинов и специфических шаперонов», регистрационный номер АААА-А20-120011790172-9.

Конфликт интересов. Коллектив авторов подтверждает отсутствие конфликта интересов по данному исследованию в ходе его проведения и на момент подачи рукописи данной статьи в редакцию, о котором следовало сообщить.

Участие авторов. Касьянова Ю.В. — существенный вклад в концепцию и дизайн исследования, получение, анализ данных и интерпретация результатов, написание статьи; Васюкова О.В., Окороков П.Л. — существенный вклад в концепцию и дизайн исследования, анализ результатов, внесение в рукопись существенной (важной) правки с целью повышения научной ценности статьи; Зураева З.Т. — получение результатов; Безлепкина О.Б. — внесение ценных замечаний. Все авторы одобрили финальную версию статьи перед публикацией, выразили согласие нести ответственность за все аспекты работы, подразумевающую надлежащее изучение и решение вопросов, связанных с точностью или добросовестностью любой части работы.
